# Bone Health and Denosumab Discontinuation in Oncology Populations

**DOI:** 10.1093/oncolo/oyac213

**Published:** 2022-10-17

**Authors:** Yee-Ming Melody Cheung, Alicia Morgans, Ole-Petter Riksfjord Hamnvik

**Affiliations:** Division of Endocrinology, Diabetes, and Hypertension, Department of Medicine, Brigham and Women’s Hospital, and Harvard Medical School, Boston, MA, USA; Department of Medicine, Endocrine Unit, Austin Hospital, The University of Melbourne, Victoria, Australia; Department of Medical Oncology, Dana-Farber Cancer Institute, and Harvard Medical School, Boston, MA, USA; Division of Endocrinology, Diabetes, and Hypertension, Department of Medicine, Brigham and Women’s Hospital, and Harvard Medical School, Boston, MA, USA

**Keywords:** bone health, cancer, denosumab discontinuation, multiple vertebral fractures, rebound phenomenon

## Abstract

Managing bone health after denosumab cessation is a commonly encountered challenge. Although the “rebound phenomenon” is generally recognized by endocrinologists in the context of osteoporosis, it is not as widely understood in the context of bone metastases and cancer. This commentary reviews the evidence on the efficacy and safety of various bone health agents in mitigating the “rebound phenomenon” in cancer populations.

Denosumab is an antiresorptive agent that is approved for the management of metastatic bone disease in patients with solid tumors and is also indicated for the prevention of bone loss and/or fragility fractures associated with various cancer therapies. It is a monoclonal antibody that binds to RANK ligand, thus preventing RANK ligand interacting with RANK receptors. An important consequence of this interaction is the inhibition of maturation of osteoclast precursors, and a subsequent promotion of apoptosis of mature osteoclasts.^1^ This results in the accumulation of osteoclast precursors and a decline in mature osteoclast number and activity.^1^ As denosumab is not incorporated into the bone matrix, its effects wear off relatively quickly post discontinuation, leading to rapid maturation of the readily available, pre-formed pre-osteoclasts, and a high bone remodeling state, termed the “rebound phenomenon”.^1^ This phenomenon has been associated with significant bone structure compromise (decreased cortical bone thickness, decreased trabecular bone volume, increased amount of unmineralized bone), high levels of bone turnover markers (BTMs) (carboxy-terminal collagen cross links (CTX), cross-linked N-telopeptide of Type 1 Collagen (NTX), procollagen type 1 N-terminal propeptide (P1NP)), and clinically manifests with multiple vertebral fractures (MVF).^1,2^ Therefore, there has been growing recognition of the risks associated with the discontinuation of denosumab.

Managing bone health after denosumab cessation is a commonly encountered challenge in individuals with cancer. However, while the “rebound phenomenon” is generally recognized and considered among endocrinologists caring for osteoporosis populations, it is not as widely understood in the context of bone metastases and cancer. This commentary reviews the evidence surrounding the efficacy and safety of various bone health agents in mitigating the “rebound phenomenon” in cancer populations.

Antiresorptive agents, such as zoledronate and denosumab, have been approved for the management of metastatic bone disease in patients with solid tumors and have been shown to reduce the development of skeletal related events (SREs) in patients with bone metastases.^3,4^ Antiresorptive agents are also prescribed for the prevention of bone loss and/or fragility fractures associated with androgen deprivation therapy (ADT) for prostate cancer and endocrine therapy for breast cancer. However, despite their proven efficacy, antiresorptive agents are underutilized in the care of patients.^5-8^ While the exact reasons for their underuse remain unclear, contributors include physician awareness and prioritization, insurance coverage, and concerns around drug adverse effects.^8^

Common adverse effects for zoledronate include systemic inflammatory symptoms, and renal toxicity.^9^ Severe adverse events associated with both agents include hypocalcemia, atypical femoral fractures, and osteonecrosis of the jaw.^9,10^ It is important, however, to recognize that in individuals at high risk of fractures/SREs, the risk of not treating with antiresorptive agents often far exceeds the risk of these rare adverse effects.

In addition to the side effects that occur during treatment, there are growing concerns regarding the risks of MVF associated with the discontinuation of denosumab. While the half-life of denosumab is relatively short (26-28 days), the time it takes for bone remodeling to resume, and for the “rebound phenomenon” to occur, is potentially longer.^10,11^ In non-cancer osteoporosis populations, denosumab discontinuation has been associated with rapid increases in BTMs within 9 months of the last denosumab dose.^12^ Similarly, the earliest development of MVF has been reported at 7 months after the last denosumab dose.^10,11^ The known predictors for MVF in this population include prior vertebral fractures^13^ and a longer duration of denosumab treatment.^14^ However, it is important to note that MVF have also been reported in individuals with no history of vertebral fractures or osteoporosis.^13,15^ This is particularly relevant to oncological populations where bone mineral density (BMD) is often normal,^2^ and MVF risk can readily be underestimated.

Furthermore, it may be difficult to distinguish the “rebound phenomenon” in individuals with cancer from the risk of bone complications related to progression of bone metastases. For example, in a study of 1414 patients with adult cancer who discontinued denosumab, several risk factors for developing SREs were noted (a shorter denosumab treatment duration, a greater number of clinic visits/hospitalizations during the denosumab treatment period, younger age at onset of bone metastases, a shorter time to denosumab initiation after diagnosis of bony metastases, and having a diagnosis of prostate cancer).^16^ Notably, SREs were not limited to vertebral fractures/MVF, but included a broad set of conditions such as spinal cord compression, pathological fractures, bone palliative radiotherapy, or bone surgery, most of which would not be expected to be a consequence of the “rebound phenomenon” alone.

The literature regarding denosumab cessation specifically in cancer survivors is limited. The only prospective data come from a phase II follow-up study of women with breast cancer.^17^ The preliminary results of this study demonstrate that in postmenopausal women with hormone receptor positive, early breast cancer treated with adjuvant aromatase inhibitor therapy,^17^ those that ceased denosumab after a median of 7 doses had significantly higher risk of developing clinical vertebral fractures over the follow-up period of 36 months compared with those who ceased placebo (HR 2.44, 95% CI 1.12-5.32).^2^ Risk of MVF was also increased, but this did not reach statistical significance (HR 3.52, 95% CI 0.98-12.64).^2^ However, whether the risk of rebound fractures extends to patients with skeletal metastases where denosumab is prescribed at higher doses has not been defined as evidence is currently limited to case reports.^18,19^

Given the association between denosumab discontinuation and the “rebound phenomenon”, the current position from osteoporosis-focused guidelines is that a denosumab drug holiday is not recommended.^20-23^ As there are well-established data demonstrating relatively low rates of adverse effects associated with up to 10 years of denosumab therapy,^24^ one option when using denosumab is to continue treatment indefinitely as life-long preventative therapy. Of note, in individuals with cancer where denosumab is administered for the treatment of bone metastases, discontinuation of denosumab is usually only considered in those with good prognostic features (ie, oligometastatic disease, a perceived low risk of bone complications and a durable response to systemic therapy).^25^ Therefore, long-term treatment with denosumab may already be implemented in a large proportion of patients with bone metastases. In contrast, among patients where denosumab is prescribed purely to protect against the detrimental impact of cancer therapies on bone loss and fragility fracture risk (ie, ADT, endocrine therapy), the duration of denosumab therapy often corresponds with the duration of the cancer therapy. As the duration of cancer therapy can vary significantly from patient to patient, lifelong denosumab may not always be an appropriate option, and choosing an alternate antiresorptive therapy or plans to support bone health in the setting of discontinuation of denosumab must be considered. Other clinical scenarios where discontinuation of denosumab should be considered include:

1. BMD improvement (antiresorptive continuation is no longer clinically indicated)
2. Patient preference (ie, adverse effects, costs, not desiring ongoing treatment)

If the decision is made to discontinue denosumab, patients should be transitioned to another antiresorptive agent.^20-23^ The optimal transition regimen has yet to be determined.^13,20,21^ Herein, we describe the various agents that have been studied in non-cancer osteoporosis populations following denosumab cessation.

## Zoledronate

Bisphosphonates (zoledronate, alendronate and risedronate) inhibit osteoclastic bone resorption by attaching to hydroxyapatite binding sites on bony surfaces. The incorporated bisphosphonate impairs the ability of osteoclasts to adhere to the bony surface and also inhibits the stimulation of bone resorption.^26,27^ Zoledronate is a potent and long-acting bisphosphonate which is administered as an intravenous infusion. To date, there is extensive evidence supporting the use of zoledronate in the prevention of SREs. This makes zoledronate an attractive option for mitigating the “rebound phenomenon” associated with denosumab discontinuation in cancer populations. For example, in a randomized controlled trial (RCT) of 643 men with castration-resistant prostate cancer and bone metastases, zoledronate was associated with a decreased rate of SREs at 15-months (44% vs. 33%; *P* = .02) and increased time to first SRE (329 days vs. >500 days; *P* = .011).^28,29^ Similarly, in a phase III study of 1648 patients with either stage 3 multiple myeloma or advanced breast cancer and at least one bony lesion, zoledronate was found to be effective and well tolerated in the prevention of SREs.^30^

With respect to the use of zoledronate post denosumab cessation, studies have been limited to non-cancer osteoporosis populations. In an RCT involving 57 postmenopausal women who achieved non-osteoporotic BMD with denosumab treatment (mean treatment duration 2.2 years), patients were randomized to either a single dose of zoledronate (*n* = 27, administered 6 months after the last dose of denosumab) or to continue denosumab 6 monthly for an additional 2 doses (*n* = 30).^31^ In the zoledronate group, women were followed for the 2 years post administration of the single dose of zoledronate, while in the denosumab continuation group the women were followed for the 1 year during which denosumab was continued and then for another year after denosumab cessation. After 2 years of follow-up, both groups saw a decline in the BMD of the lumbar spine (LS) from peak levels. However, in the group that received an additional year of denosumab treatment, the LS BMD declined to lower than pre-denosumab levels, while in the zoledronate group, it decreased back to pre-denosumab levels. Therefore, a single infusion of zoledronate given 6 months after the last denosumab dose, does attenuate bone loss for at least 2 years. Furthermore, in the zoledronate group, shorter duration of denosumab treatment increased the likelihood of maintaining BMD after zoledronate administration. Individuals who had received a total of ≤6 denosumab injections demonstrated a maintenance of both LS and femoral neck (FN) BMD at 1 year (12 months from time of zoledronate administration), while LS and FN BMD decreased in those with >6 injections over the same time period.^32^

In a 2-year RCT, the effect and timing of zoledronate administration post denosumab discontinuation was evaluated in postmenopausal women and men ≥50 years with osteopenia (*n* = 61), who had received a single dose of zoledronate after denosumab (mean denosumab treatment duration 4.6 years).^33^ In one group, the zoledronate was administered at 6 months post denosumab discontinuation, in the second group zoledronate was administered at 9 months post denosumab discontinuation and in the third group zoledronate was administered at 12 months post denosumab discontinuation, or if CTX rose above 1.26 μg/L or if BMD decreased by >5% prior to 12 months. Each treatment group lost BMD at all sites between the time of denosumab discontinuation and the 2-year follow-up (no between-group differences). The study concluded that irrespective of the timing, zoledronate treatment did not fully prevent BMD loss in patients with osteopenia following denosumab discontinuation.^33^

## Alendronate

Alendronate is a bisphosphonate that has the added benefits of being orally administered, making it a relatively convenient option in the treatment of the post-denosumab “rebound phenomenon”. Furthermore, several studies have demonstrated the benefits of alendronate on BMD in cancer populations. One such study was performed in individuals with prostate cancer. In this retrospective cohort study, 47 men with prostate cancer who received ADT and had ≥2 BMD measurements (baseline and follow-up) were identified. Twenty-two patients received weekly alendronate during the follow-up period (17.6 ± 8.3 months), while 25 did not receive any antiresorptive therapy. There was a greater loss in BMD per year at the LS (–1.29 ± 0.7% vs. +1.41 ± 0.7%), total hip (–0.94 ± 0.6% vs. +0.97 ± 0.5%), and FN (–2.17 ± 0.7% vs. +0.32 ± 0.6%) in the patients not treated compared with those treated with alendronate.^34^

The use of alendronate post denosumab cessation has also been limited to data from non-cancer osteoporosis populations. In a post hoc analysis of a 2-year RCT, 126 postmenopausal women (mean age 65 years) without cancer who had received 2 doses of denosumab for the treatment of osteoporosis were transitioned to once-weekly alendronate. Alendronate was commenced 6 months after the second dose of denosumab and was continued for a total duration of 12 months. Alendronate for 12 months appeared to either maintain or increase BMD at the LS and hip following denosumab discontinuation.^35^ A limitation of this study was that denosumab was only administered for 1 year. With longer durations of treatment there is a greater potential for bone loss post treatment cessation, and therefore it may be more difficult to preserve bone mass when transitioning to alendronate.

## Risedronate

Risedronate is an oral bisphosphonate which has also been shown to be safe and efficacious in preventing BMD loss in both breast and prostate cancer populations. In a 2-year RCT, 87 postmenopausal women with breast cancer were randomly assigned to weekly risedronate or placebo.^36^ At 2 years, women in the placebo group had significant (–1.4% to –2.4%) loss of BMD at all sites while BMD remained stable at the LS and hip in the risedronate group. Similarly, in a 2-year RCT conducted in 104 patients with nonmetastatic prostate cancer receiving radiotherapy plus ADT, patients randomized to weekly risedronate had less BMD loss at 2 years and also had a significant suppression of bone turnover biomarkers for 24 months when compared with controls.^37^

There are limited studies investigating the effects of risedronate post denosumab, and data remains limited to non-cancer osteoporosis populations. In a follow-up of the Fracture Study in Postmenopausal Women with Osteoporosis (FRAME) study, 19 patients who received romosozumab or placebo for 1 year, followed by denosumab for 2 years were transitioned to either zoledronate, risedronate or no treatment (based on patient preference).^38^ Five participants received risedronate, 11 received zoledronate (given 8 months after the last dose of denosumab) and 3 did not receive any therapy. One year after the transition, both risedronate and zoledronate groups demonstrated retention of treatment effect on BMD. However, those receiving risedronate had greater bone loss at both the LS and hip when compared with those receiving zoledronate. In comparison, the individuals who did not receive any follow-up treatment demonstrated a loss of 80%-90% of the BMD gained during the FRAME study.

## Other

There is currently a lack of quality evidence for the safety and/or efficacy of raloxifene, teriparatide/abaloparatide, and romosozumab in the use of patients with or without cancer. Therefore, these agents are currently not recommended as transitional regimens post denosumab in cancer populations.

**Figure 1.**
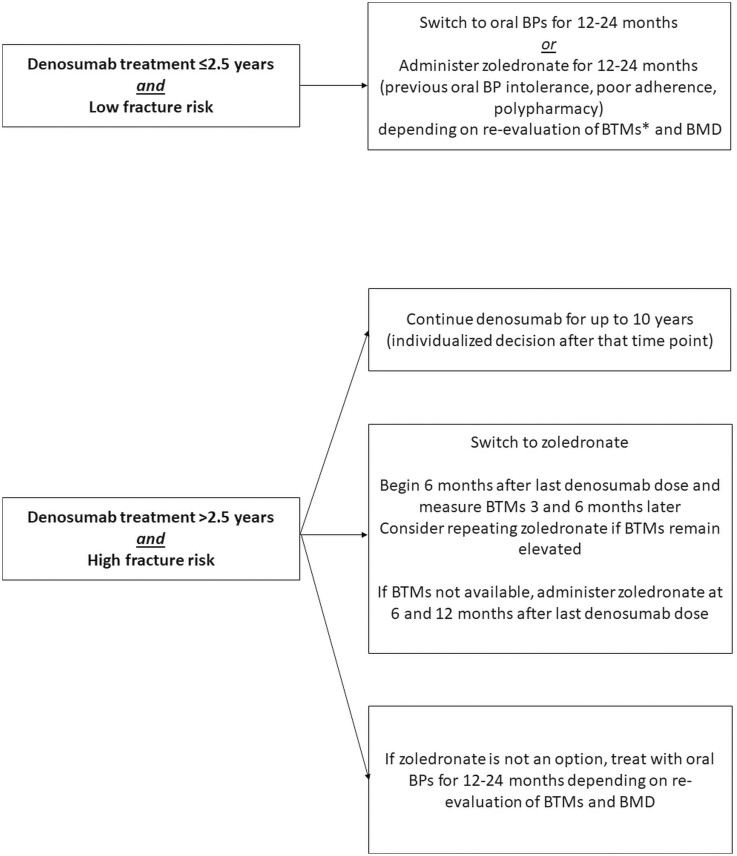
Adapted from the ECTS Guidelines for denosumab discontinuation. ECTS Guidelines for denosumab discontinuation.^23^ ^High fracture risk is defined as having risk factors linked to prevalent osteoporosis fractures and concomitant comorbidities (eg, continuous use of glucocorticoids or aromatase inhibitors, diabetes, inflammatory diseases, frailty, etc.).^23^ *BTM targets post bisphosphonate suggestive of an adequate response include CTX <280 ng/L or P1NP <35 μg/L. Bisphosphonate (BP), bone mineral density (BMD), and bone turnover markers (BTMs).

Although the current evidence for the various denosumab transition regimens is limited, the European Calcified Tissue Society (ECTS) has recently issued guidelines for denosumab discontinuation follow-up therapy in non-cancer patient populations with osteoporosis (Fig. 1).^23^

Oncology societies have yet to provide formal guidance regarding denosumab discontinuation. While it would be reasonable to extrapolate many of the ECTS recommendations to oncology populations, it is important to be aware of potential caveats where aspects of the ECTS guidelines may not be appropriate (ie, the BTM targets may not be applicable for individuals with a high burden of skeletal metastases).

In our current practice, we attempt to consider the rebound phenomenon of denosumab discontinuation when making treatment decisions about antiresorptive agents in patients with cancer. In patients who are expected to need a definitive period of treatment to reverse osteoporosis, we generally favor the use of bisphosphonate therapy. However, as denosumab is associated with greater continued and progressive increases in BMD when compared with bisphosphonates, we would consider denosumab in patients with severe osteoporosis, or in those who remain osteoporotic despite treatment with bisphosphonates. Among patients who have been treated with more than 2 doses of denosumab, we attempt to avoid denosumab discontinuation. However, if denosumab cessation is necessary, we generally administer intravenous zoledronate 6-8 months after the last injection of denosumab, followed possibly by a second dose of zoledronate if BTMs are elevated 3-6 months after the initial dose of zoledronate has been administered.

In summary, antiresorptive therapies play a central role in the prevention of fragility fractures as well as SREs in individuals with cancer. Therefore, as long as the complications associated with treatment and treatment cessation are considered, it remains essential to use these agents where appropriate. However, as the discontinuation of denosumab is not completely benign, a greater awareness among physicians and oncology communities is needed regarding the associated risks of the “rebound phenomenon” and MVF. Although the current evidence for denosumab transition regimens is based on individuals with osteoporosis, these proposed strategies can be used as a guide for the management of oncology populations during a time of limited evidence.
